# Lifetime Estimation and Orientation Effect Based on Long-Term Degradation Analysis of Thermoset and Thermoplastic Insulators

**DOI:** 10.3390/polym14193927

**Published:** 2022-09-20

**Authors:** Israr Ullah, Rahmat Ullah, Muhammad Amin, Rahisham Abd Rahman, Aftab Khan, Nasim Ullah, Sattam Alotaibi

**Affiliations:** 1 Faculty of Electrical Engineering, Ghulam Ishaq Khan Institute of Engineering Sciences and Technology, Topi, Swabi 23460, Pakistan; 2 Faculty of Electrical and Electronic Engineering, University Tun Hussein Onn Malaysia, Batu Pahat 86400, Malaysia; 3 Department of Computer Science, Allama Iqbal Open University, Islamabad 44310, Pakistan; 4 Department of Electrical Engineering, Taif University, Al Hawiyah, Taif 26571, Saudi Arabia

**Keywords:** polymeric insulators, aging, SiR, TPE, FTIR, leakage current, life estimation

## Abstract

Polymeric insulators have replaced ceramic insulators due to their obvious properties like low surface energy, which exhibits good hydrophobic performance, low weight, etc. However, electric utilities have concerns about their long-term performance. In that context, the long-term performance of two different types of polymeric insulators are investigated in this study: thermoset Silicone rubber (SiR) and thermoplastic elastomeric (TPE). Multi-stress aging was performed in the different orientations of both types of polymeric insulators. During multi-stress aging, insulators are exposed to varied loads in both vertical and horizontal orientations, simulating actual service environmental conditions. Experiments were done in a chamber where different types of stresses were simulated, which resembles the weathering conditions of Hattar, Pakistan, which is one of the most polluted industrial zones. Both insulators were stressed in a chamber under the designed weathering conditions for two years and six months at different orientations. Polymeric insulators made of SiR perform better in the vertical position than that in the horizontal position. Furthermore, the experimental results show that both materials are capable in a variety of situations. SiR, on the other hand, performed well due to its high hydrophobicity, which means it is less impacted by contaminants and hence has a longer life and higher service performance than TPE.

## 1. Introduction

Insulation is a key feature of high-voltage transmission systems, contributing significantly to system reliability. Due to the inherent benefits of polymeric insulators over traditional ceramic and glass insulators, it is nowadays increasingly being employed for outdoor insulation. However, electric utilities have concerns about their long-term performance, and their properties must be assessed before their deployment in the field [1,2]. Therefore, a new type of technique is used called multi-stress aging, which is performed in a designed, simulated chamber. A weather cycle for a specific area is designed and then applied in a chamber. Schneider was the first to use a multi-stress aging concept in an accelerated technique for insulators research in 1991 [1,2]. Different types of stresses [2,3,4,5,6] are applied at the regular interval following the designed weather cycle [7,8].

Insulating polymers must be thoroughly examined before installation due to their biological origins and varying performance in diverse environments. One of the most effective approaches for testing the performance of polymeric insulators is multi-stress aging. Different types of multi-stress aging experiments have been performed till now on different polymeric materials [9,10,11,12,13]. However, mostly they are performed for a short interval of time which does not predict the actual life expectancy of polymeric insulators. Among polymeric insulators, silicone rubber performs well in diverse polluted conditions due to its low surface energy [14,15]. As a result, it suppresses leakage current and decreases the chances of flashover occurrences [16,17].

In [18], the authors performed the aging study of thermoplastic and silicone rubber by simulating the weathering cycle of Mexico study for 2544 h. They observed that silicone rubber outclassed thermoplastic insulators. Venkatesulu et al. [19] analyzed the aging of full-scale distribution class silicone rubber composites insulators and detected a loss in low molecular weight (LMW) molecules. Furthermore, surface roughness was also noticed. The authors in [20] conducted a 5000-h aging test on Silicone rubber (SiR) insulators. They initiated a new aging technique known as the dip method. They concluded that the leakage current is solely determined by the insulator’s surface state. Another research examines how ultraviolet (UV) weathering affects ethylene propylene diene monomer rubber (EPDM) insulators’ morphological, chemical, and thermal characteristics. The insulators aged due to oxygenated species and EPDM [21] structural depolymerization. Furthermore, authors in [22] used Inclined Plane Tracking (IPT) and Erosion experiments to analyze SiR insulators. They use the recurrent plot (RP) to examine the aged samples. They characterized SiR insulators using Scanning electron microscopy (SEM), Energy dispersive X-ray analysis (EDAX), and Fourier transform infrared analysis (FTIR) and found that the RP technique might be utilized to analyze aged materials. Multiple stressors resulted in the decline in hydrophobicity of the samples were observed which were due to the multiple stresses [23]. Similarly, the review paper [1] discusses the impact of different stresses on the performance of SiR insulators.

Xidong et al. [24] proposed an optimized aging test method for polymeric insulator performance under high and extra-high voltages with minor changes to the IEC 5000 h test standard. The long-term onsite aging behavior of silicone rubber insulators was investigated. The reliability of the aforementioned insulators was investigated by examining changes in hydrophobicity with aging, erosion, and tracking resistance. A new multi-stress aging method was also presented to assess the performance of nanocomposite insulators for long-term applications. Moreover, the effectiveness of 11 kV full-scale polymeric insulators was explored over a one-year period using natural aging. The performance has been evaluated using cutting-edge methodological approaches such as SEM and Fourier Transform Infrared (FTIR) spectroscopy [25].

Even though numerous research groups worldwide have conducted a great deal of study, there is still a need for a thorough examination of polymeric insulators’, especially in a given environment, before installation. In this study, insulators made of SiR and TPE materials were aged under several stresses for 21,504 h. Techniques for both theoretical and practical material analysis were employed to evaluate the findings. This study compared SiR and TPE under identical stress settings to compare their long-term performance as insulators in polluted industrial areas. The experimental set-up of the environmental chamber and the weather cycles created for the aging of insulators are shown in Section 2.

## 2. Methodology

In the High Voltage Research (HVR) lab, insulators were subjected to accelerated multi-stress aging. Meteorological data of the industrial region Hattar was collected over the last 30 years from Pakistan Meteorological Department (PMD) [26,27]. A systematic weather cycle consisting of both winter and summer cycles was designed based on the data collected from PMD representing the environment of Hattar, Pakistan, through rigorous calculations, as shown in Table 1. Each parameter was calculated for better design of the environment.

Most researchers have conducted aging for the utmost 5000 h and very few studies are available for maximum durations of aging. Therefore, for this study, the maximum duration was selected to elucidate the better aging performance of the polymeric insulators. Moreover, the accelerated aging was done for the duration of 21,504 h, which represents 32 years of real service. After each summer and winter cycle, samples were obtained from the insulators for examination. Analyses procedures included visual observations of materials, hydrophobicity classes, FTIR analysis, and leakage current measurements.

## 3. Experimental Set-Up

**Figure 1:**
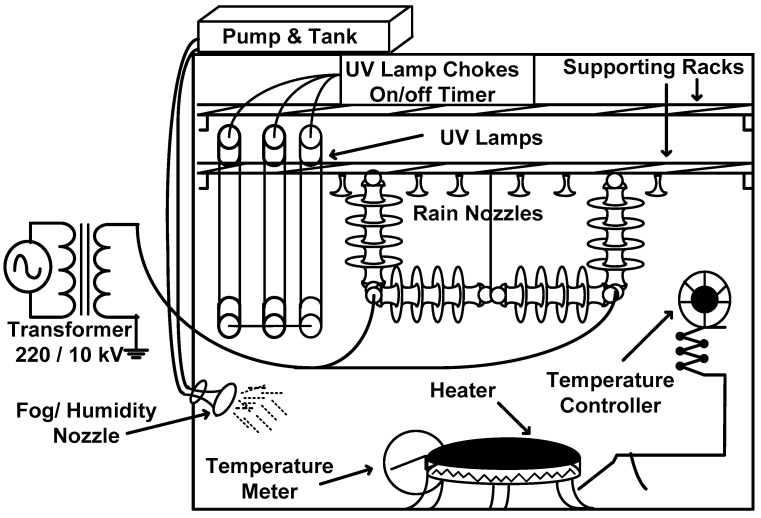

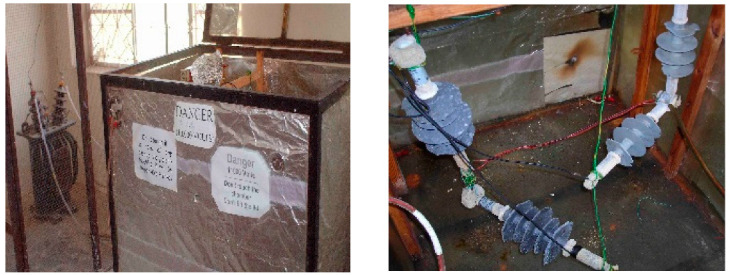
Schematic & photographs of the experimental setup.

**Figure 2:**
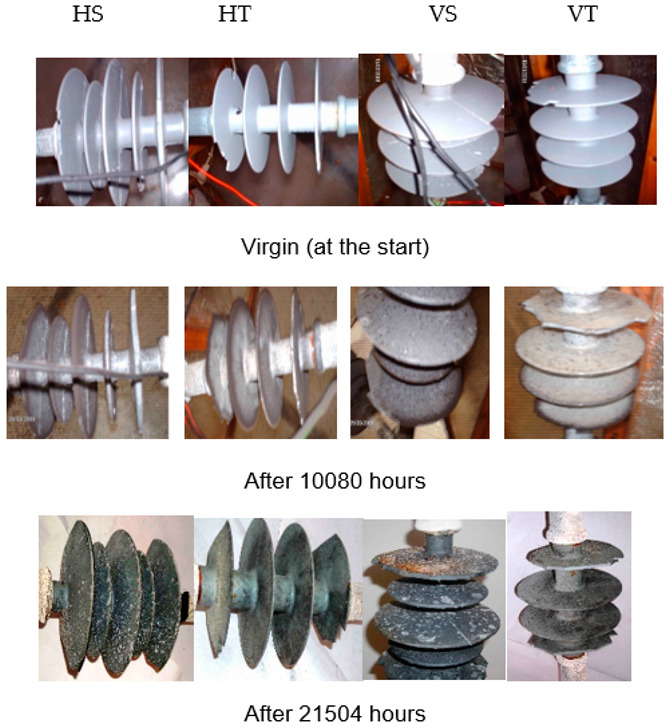
Results of visual observation.

This experiment used a one-meter-diameter environmental glass chamber with a one cubic meter volume. A 10 kV (L-N) transformer, as well as regulated UV, were included in this room to stress the insulators. A light, leakage current monitoring setup, controlled heating system, rain and humidity generation/salt fog setup, nozzles (50 × 1 mm), and pump were installed in the chamber. Figure 1 and Figure 2 show the schematic diagram and real-time pictures of the environmental chamber, respectively.

Two TPE and two SiR full-scale insulators (for a total of four) having creepage lengths ~530 mm were put in horizontal and vertical configurations. The insulators were given the following names:

**HS:** SiR Insulator with a Horizontal Orientation
**VS:** SiR Insulator Oriented Vertically
**HT:** TPE Insulator, Horizontally Oriented (HT)
**VT:** TPE Insulator, Vertically Oriented (VT)

Two small pieces were cut from each test sample from the top and bottom ends and labeled with D and E, respectively.

The insulators were initially investigated through visual observation using a magnifying glass for any apparent surface changes like macro cracks and pollution deposits. To evaluate the surface hydrophobicity variations with aging, the Swedish transmission research institute (STRI) classification procedure was utilized. According to the STRI guide, the test samples are sprayed with tap water, and the surface images are taken with a high-resolution camera. A hydrophobicity class is assigned to the surface after comparing the photographs with the standard image provided in the STRI guide, as discussed in [28,29]. The STRI classification is widely used as it is more handy and efficient where surface hydrophobicity estimation of several samples after each distinct aging cycle is required [30,31]. Moreover, changes in functional groups and morphological characteristics were analyzed by utilizing Fourier transform infrared spectroscopy (FTIR) and Scanning electron microscopic (SEM) images.

## 4. Results and Discussions

### 4.1. Visual Interpretations

Visual interpretation of all the samples was made at the end of 10,080 h and the end of the aging period, i.e., after 21,504 h. Some of them are shown in Figure 2.

The equivalent salt deposit density (ESDD) and Non-soluble Deposit Density (NSDD) readings were based on tests conducted every 672 h following International Electrotechnical Commission (IEC) IEC 60,815 standards [32]. To calculate ESDD, insulators were firstly managed to wipe, and solution conductivity was evaluated with a Total Dissolved Solids TDS meter-3. After drying, the difference in weight of the filters was measured using a weight scale to obtain the NSDD results.

**Figure 3:**
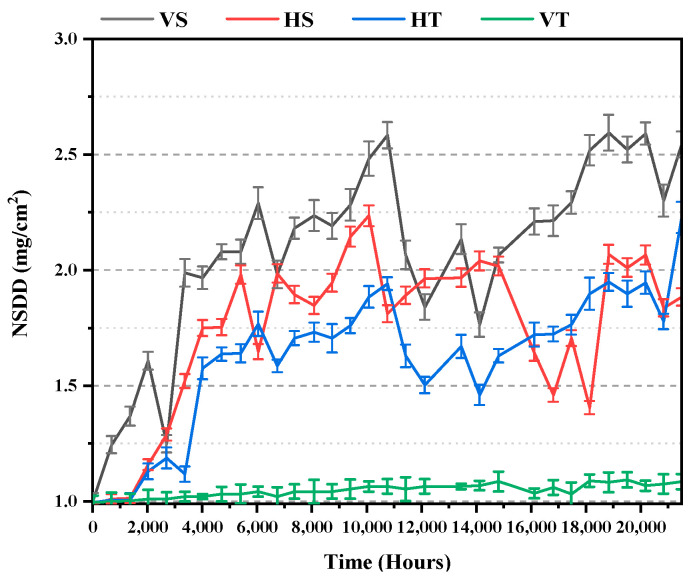

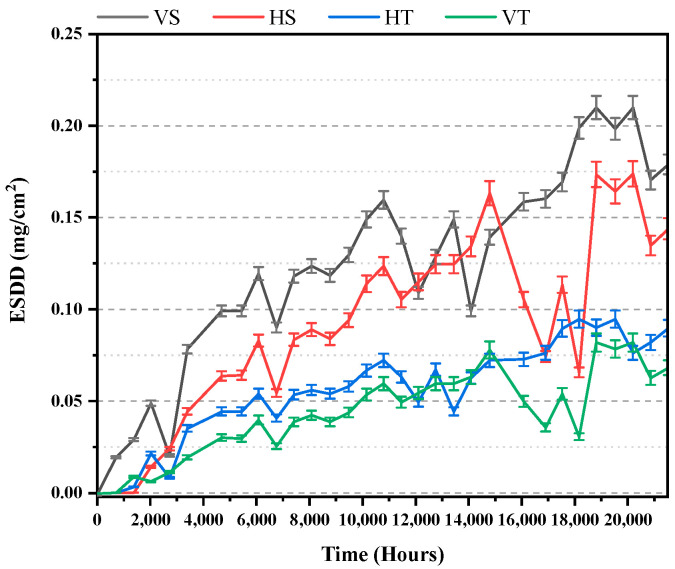
ESDD & NSDD measurements.

Vertical insulators have higher salt deposits and discoloration effects than horizontal insulators. It was most likely because there was less rain to clean the insulators, resulting in higher salt accumulation due to salt fog. TPE insulators had a higher degree of discoloration than SiR insulators. Figure 3 depicts a plot of non-soluble deposit density (NSDD) and equivalent salt deposit density (ESDD) values. The presence of carbon was also noticed on the surface of test samples at the end of 10,080 h; however, it extinct slowly afterward at 13,440 h. Interestingly, it resurfaced after 18,816 h and eventually went away. As indicated by the findings of different methodologies in the following sections, this signifies the reversible change in these materials at those locations.

### 4.2. Hydrophobicity

**Figure 4:**
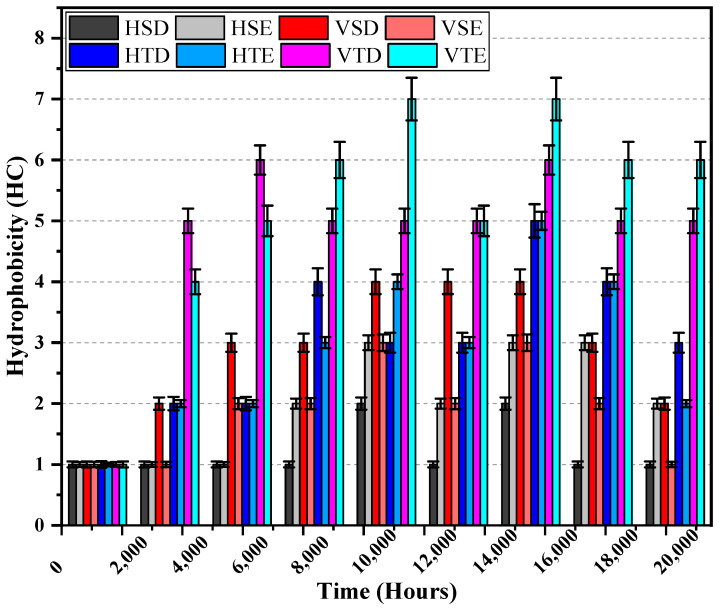
Hydrophobicity classification.

The Swedish Transmission Research Institute (STRI) was employed to find the hydrophobicity class (HC) of the samples after each summer and winter cycle [33]. Figure 4 shows the data gathered at various points throughout the investigation.

According to the results of the experiment, SiR insulators maintained superior hydrophobicity, but TPE insulators lost hydrophobicity quickly. In 10,080 and 18,816 h, insulators lost their hydrophobicity abruptly. At the same time, a blackish chalky look was discovered. In terms of hydrophobicity, all horizontal insulators outperformed their vertical counterparts, achieving exceptional hydrophobicity. Impurities are washed out more in horizontally oriented insulators than in vertically oriented insulators, which is why the following circumstances exist. In comparison with energized ends, HC declines more for the lower ends of the insulators. This may be attributed to the diffusion of low molecular weight (LMWs) from the bulk of the insulator to the surface, and also, it doesn’t occur at the lower end [33,34].

### 4.3. Current Leakage

**Figure 5:**
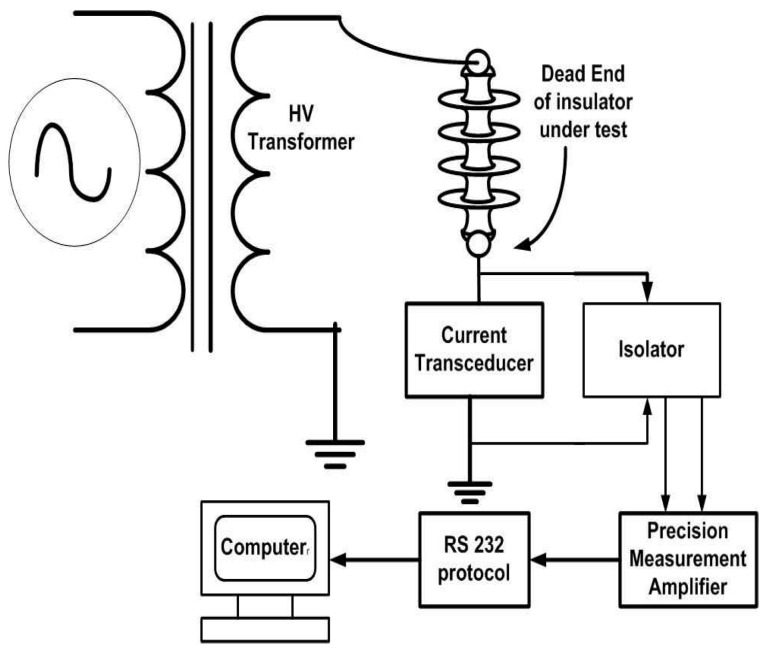
Setup for measurement of leakage current.

**Figure 6:**
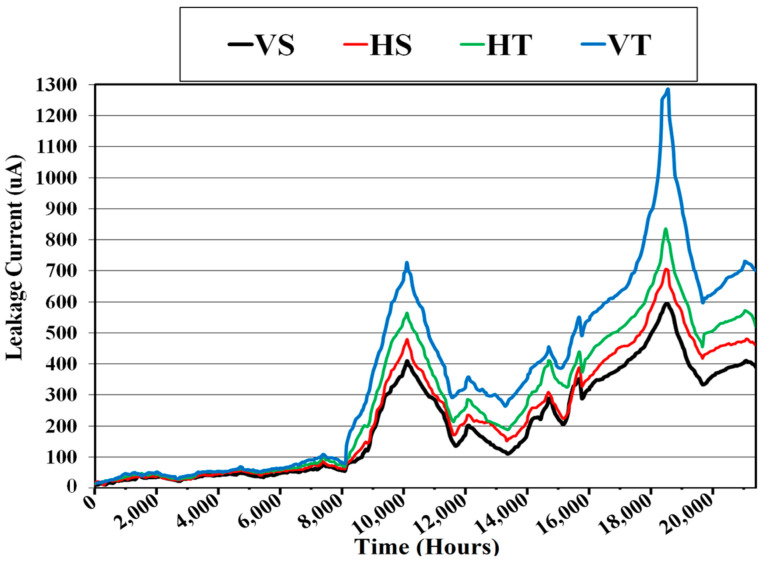
Leakage current variation.

Leakage current (LC) was measured simultaneously during the study. A series one-ohm resistor was used to measure LC between the ground and dead ends of the insulators. The current through the linked computer was calculated using the voltage across each resistor. Figure 5 shows a schematic of the created configuration. Figure 6 depicts the leakage current results.

The greatest peak magnitude of leakage current in vertical TPE insulators was measured throughout the experiment. The same sample has a lot of discoloration and hydrophobicity loss. Vertical SiR insulators had the lowest leakage current values. The results demonstrate a progressive rise in leakage current, following the degradation and recovery processes. It rises in comparison at first, then falls, but each cycle’s values are somewhat higher than the preceding one, resulting in an overall rise. The self-recovering nature of polymers causes LMW molecules to migrate from bulk to surface, resulting in the behavior described above.

Leakage currents are particularly strong between 10,080 and 18,816 h compared with other times. The findings are consistent with both the hydrophobicity categorization and visual inspection.

### 4.4. FTIR

**Figure 7:**
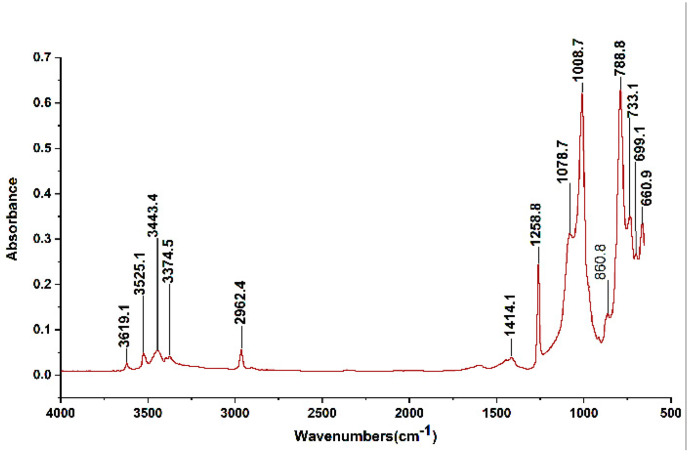
A typical FTIR absorption spectrum.

The variation in the structure of polymers was investigated using Fourier transform infrared analysis (FTIR). The FTIR spectrometer model, namely Perkin Elmer Spectrum 200, was used to create the spectrographs. Over the infrared frequencies, absorption spectra for wavenumbers between 600 cm^−1^ and 4000 cm^−1^ were recorded. Figure 7 depicts a typical FTIR absorption curve. The *x*-axis wave number corresponds to the compound inside the material. Essential components in SiR and TPE materials correspond to the values at wavenumbers 914, 1014, 1258, 2917, and 3525 [35,36]. In the main chain of polydimethylsiloxane (the foundation polymer for silicon rubber), Si-C and Si-O-Si bonds with wavenumbers of 788 cm^−1^ and 1008 cm^−1^ are significant. In contrast, C-H bonds with wavenumbers of 1260 cm^−1^ and 2962 cm^−1^ are preferred in the side chain [37].

**Figure 8:**
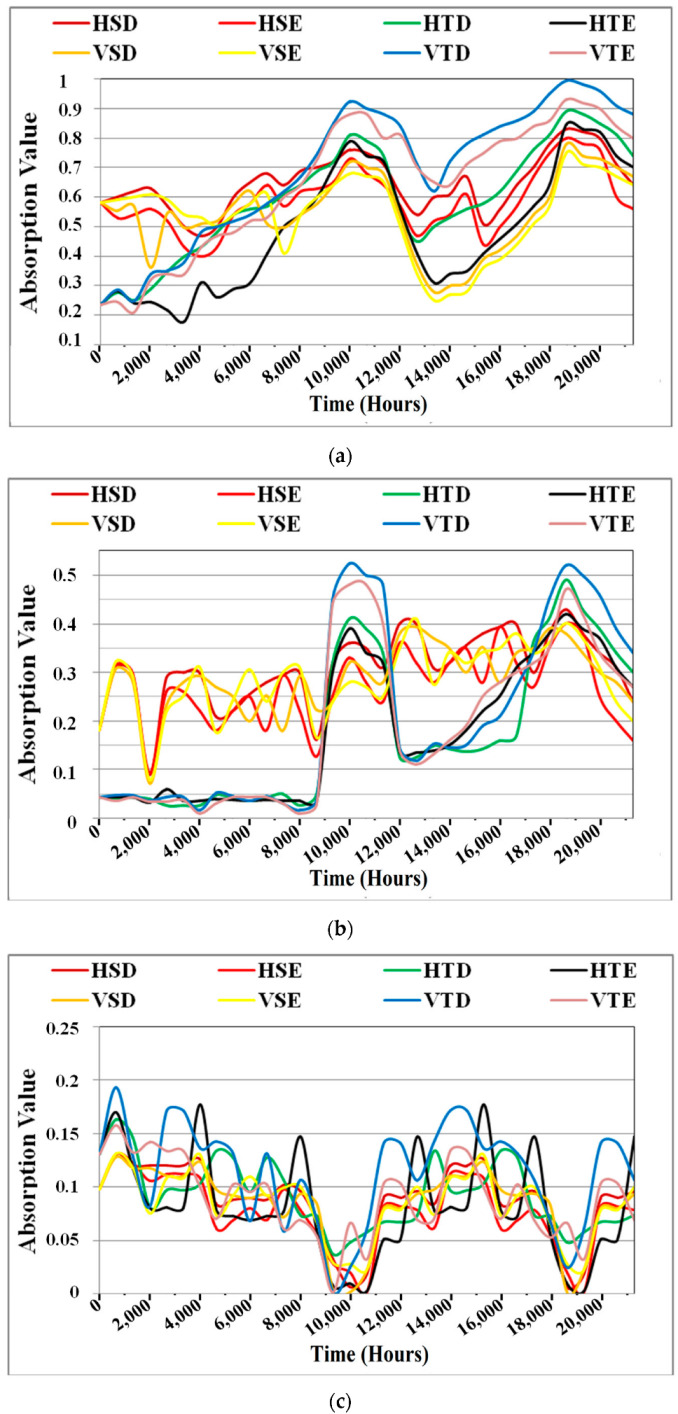

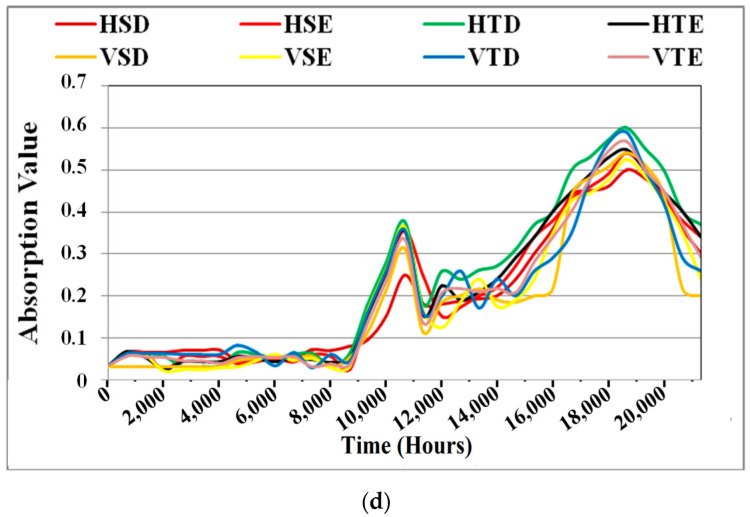
Variation of absorption values at different wavenumbers. (**a**) Absorption values variation at wave number 1014. (**b**) Absorption values variation at wave number 1258. (**c**) Absorption values variation at wave number 2917. (**d**) Variation of absorption values at wave number 3525.

The relative characteristics of IR absorption bands (wave numbers) and their associated chemical groups are summarised in Table 2 [38,39]. Because O-H stretching, CH_3_ bending and stretching, C-C-C chain stretching, and Si-O-Si stretching in polymers are of interest to us (SiR and TPE Insulators). This is why the FTIR analysis of the pertinent peaks in this study was chosen. Before the aging began, FTIR absorption peaks were measured and shown in Figure 8a–d at o hour on the *x*-axis. Changes in the absorption peaks are shown for both the aged and virgin samples in Figure 8a–d for comparative analysis. The FTIR data showed no significant change at wavenumbers 660, 698, 729, 861, 914, 1465, 1737, and 3392.

A rapid rise is observed for all the test specimens at wave numbers 788, 968, and 1014 for the time instant 10,080 and 18,816 h. Wave number 1014 is important because it correlates with the main chain Si-O-Si. At the time instant 10,080 and 18,816 h for both types of insulators under consideration, severe changes were noticed at wave number 1258, representing three carbon atoms C-C-C inside the bulk of the polymer. This wavenumber is critical since it serves as the foundation for these materials. After the conclusion of 10,080 h and 18,816 h, there is also visible chalking. This demonstrates that surface chalking has a direct impact on the absorption values of C-C-C and Si-O-Si bonds, among other things. Because the carbon strength to hydrogen link is influenced by differences in the strength of the C-C-C bond, wave numbers 1465 and 1258 behave similarly.

A wave number 2850, TPE insulators only grow at a consistent rate. This shows that the stretch of CH-2 in SiR insulators is unaffected by any load. Furthermore, TPE insulators have efficiently enhanced their flexibility and exhibit no indication of chalking after 10,080 and 18,816 h. This research demonstrates that the CH-2 bond is unaffected by surface chalking in both SiR and TPE insulators.

Wave number 2917 shows asymmetric stretching of CH_3_, which is connected to the process of surface chalking. Wave 2917, on the other hand, indicates a decrease in values between the hours of 10,080 and 18,816. A wavenumber 2917, the chalking index increased as the absorption values decreased, as illustrated in the next section. The same rising pattern with an identical ascent was witnessed at wave numbers 3430 and 3525, and the instants of 10,080 and 18,816 h. Major fluctuations in all key wave numbers were seen at 10,080, and 18,816 h, which perfectly conform with the results of all analytical approaches, which show significant declination in the insulator’s surface. These big changes will be investigated further when the anticipated service life of these insulators is computed in the next section.

### 4.5. Chalking Analysis

Surface chalking was determined by comparing the FTIR spectral peak heights of the Alumina Tri Hydrate (ATH) filler at 1020 cm^−1^ to the polymer at 2917 cm^−1^ [3,5,40]. This was done to measure the amount of chalking on the surface by scaling the relative quantities of filler and polymer. The chalking index is the name given to this ratio.

**Figure 9:**
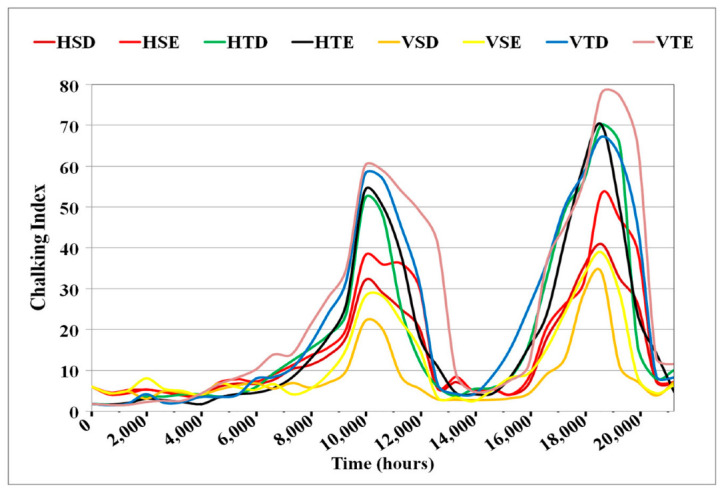
Chalking index.

C_i_ = P_f_/P_p_

where C_i_ is the chalking Index, P_f_ is the peak value of ATH at 1020 cm^−1^, and P_p_ is the peak value of polymer at 2917 cm^−1^. Figure 9 depicts the plots of chalking indices. Insulators have crossed the total normal value of 20 at two periods. The visual examination at these sites implies that insulators were also severely chalked at these times. It has now been recovered as a result of further aging.

### 4.6. Life Estimation by Regression Analysis

**Figure 10:**
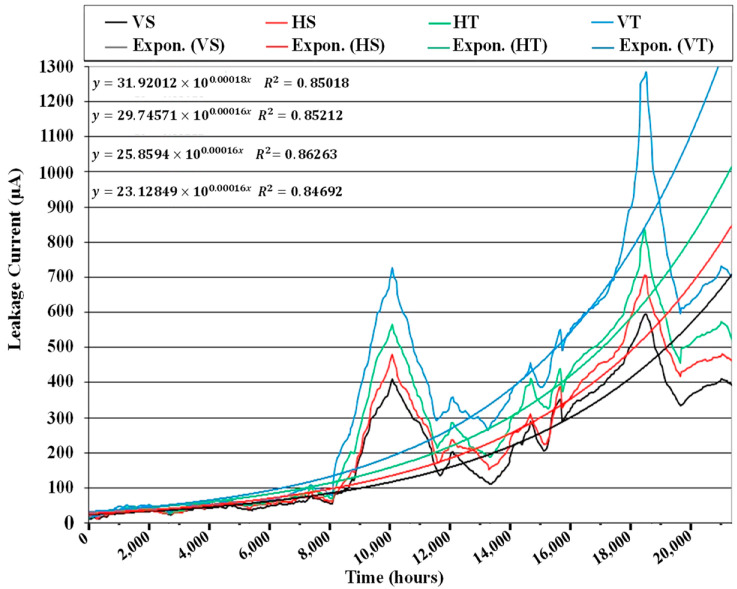
Exponential regression on results of leakage current.

The regression approach is commonly used for forecasting and life prediction. Researchers feel that the most trustworthy criterion for assessing the overall deterioration of any ceramic or polymeric insulator is LC. Different techniques, such as polynomial, exponential, and logarithmic regression, were used to fit the LC data. The fitting curves obtained from the exponential technique are shown in Figure 10. As depicted in Figure 10 that the least square value (R^2^) was found for each technique, and after that, R was calculated and displayed in Table 3.

If the value of R is 1, then it indicates that the considered model fully netted the data. Otherwise, if it is greater, then it shows overfitted model. R values close to and less than one are thus desired. When looking at table III, it is evident that the exponential model best fits the data. As a result, the exponential model will provide us with the most realistic insulator life.

**Figure 11:**
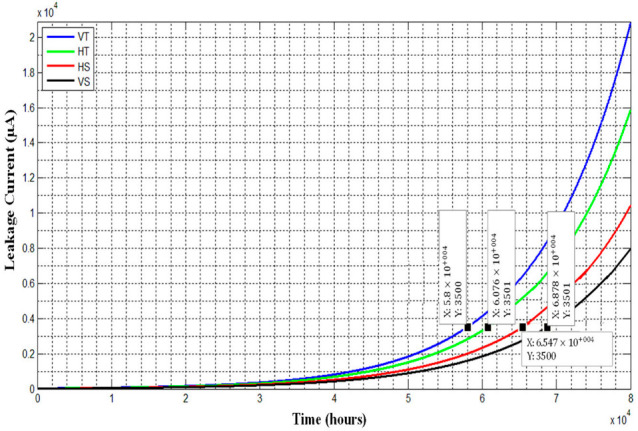
Extrapolation on results of exponential regression.

Until recently, no international standard has set a leakage current limit for transmission line insulators. Only the values for indoor insulating equipment are governed by the International Electrotechnical Commission (IEC950) and IEC16001 standards. As a result, such a limit is only applied to exterior insulators. This is, of course, a stringent requirement for outdoor insulators. However, the benefit of doing so is that we can confidently assert that if outdoor insulators have the leakage current levels indicated for inside appliances, they are trustworthy in the period before breaching this limit. The maximum current restriction for fixed indoor type devices is now 3.5 mA, according to IEC950 specifications. The time until the insulators breach the 3.5 mA limit is calculated by graphing the equation of the exponential curve derived from regression analysis, as illustrated in Figure 11.

By dividing the value of X in Figure 10 by 21,500 h and multiplying by nine simulated years, the real expected life in years can now be computed. Table 4 displays the outcomes.

Curve fitting approaches cannot capture the significant increase in the leakage current value at the end of 10,080 and 18,816 h of aging, but it is thankfully good because we know from our studies on these insulators that these points do not represent permanent insulator behavior because the insulators themselves emerged from these high current peaks without any maintenance/cleaning or relaxation durations. Because no term has been given to this phenomenon, the author names their saturation points.

The rate of rising leakage current changes after each saturation point, as seen by the current charts. The rate of increase of leakage current before the initial saturation threshold was 5 µA/year, but after that, it increased to 57 µA/year. We can explain these saturation points chemically. The degeneration of SiR and TPE materials is influenced by their hydrophobicity, which is further influenced by LMW molecules in their surface and bulk. The surface loses LMWs as degeneration begins, but a few LMWs manage to migrate from the interior mass of the material to the surface, reversing some of the degeneration. So the phenomenon of cyclic degeneration/recovery occurs. Material, on the other hand, quickly depletes all of its LMWs. As indicated in this study, at 10,080 and 18,816 h, this eventually displays a high/rapid deterioration to undesirable levels. Gaining this much surface degeneration results in a significant energy differential between the interior bulk and the surface, causing some High Molecular Weight Species (HMWs) to break down into LMWs. The same LMWs then travel to the surface, where they recover to acceptable levels. The inner bulk’s (HMWs) high molecular weight components are sufficient to survive the insulator’s mechanical life.

These saturation thresholds have little bearing on life expectancy; they only signal when maintenance or cleaning may begin. Such maintenance is optional and is dependent on the utility’s requirement to keep them running without tripping the line/flashover.

## 5. Conclusions

The aging of the two most prevalent polymeric insulator types for high voltage outdoor insulation is addressed in this study. The results revealed that electric stress influences both increase in leakage current on the insulator surface and the chemical degradation of insulating materials. However, the effect of electric stress on material hydrophobicity retention is optimistic. SiR insulators captured more pollutants than TPE insulators, according to the study. The performance of SiR relies on their hydrophobicity. SiR insulators are less affected by pollutants due to their excellent hydrophobic nature, resulting in longer life and higher service performance. TPE insulators, on the other hand, are less hydrophobic. Therefore washing and surface contamination have a greater influence. TPE insulators in the current investigation showed a reduction in hydrophobicity at an early stage. However leakage current values were within acceptable limits. In comparison to horizontal orientation, SiR insulators behaved better vertically. The reason for this is that rain washes LMWs quickly in a horizontal direction, reducing their hydrophobicity. In a vertical orientation, these insulators become more hydrophobic and even turn contaminants on them hydrophobic. Because their performance is more dependent on surface pollution, which is easily washed away in this location, TPE insulators perform better in the horizontal direction.

In SiR, the recovery phenomena occurred in which the diffusion of LMW from the bulk to the surface and hence retrieve the performance of the surface. This may be the major reason for the low degradation occurring in the case of SiR as compared with the other. Furthermore, the study confirmed the statements of many authors that SiR materials for outdoor electrical insulation are more stable and have a longer life than TPE and other carbon-based polymers. This is because the Si-O-Si bond in SiR is significantly stronger than the C-H-C bond in all TPE materials. In summary, SiR insulators have a bright future ahead of them and are also less expensive than TPE insulators. TPE insulators, on the other hand, will expand in scope due to their recyclable nature, environmental friendliness, simplicity of adding fillers, and wide range of chemical compositions, which, when applied, will undoubtedly cut costs, and improve performance.

## Figures and Tables

**Table 1:** Aging Cycles of Hattar, Pakistan.

Item	Winter (October–April)	Summer (May–September)
UVA radiations	8 h	10 h
Length of a cycle (days)	17	11
Temperature (°C)	35.3	47.2
Salt fog (6000 μS/cm)	4 times	0
Acid rain (4.5 pH)	2 times	6 times

**Table 2:** Wave Numbers in Functional Groups.

Numbers of Waves	Functional Groups
3430–3525	-OH
3392	SiOH stretching
2917–2922	Stretching-CH_3_
1465–1471	Asymmetric bending of CH_3_
1258	C-C-C
1014	Si-O-Si stretching
788–792	Si-O of Si(CH_3_)_2_

**Table 3:** Values of R taken from regression techniques.

Sample Designation	VT	HT	HS	VS
From logarithmic	0.698	0.716	0.709	0.702
From quadratic	0.872	0.889	0.890	0.876
From exponential	0.923	0.924	0.929	0.921

**Table 4:** Estimated Life.

Sample Name	Hours to Reach 3.5 mA	Estimated Life (Years)
VT	58,000	24.2
HT	60,760	25.4
HS	65,470	27.4
VS	68,780	28.7

## References

[1] Akbar M., Ullah R., Alam S. (2019). Aging of silicone rubber-based composite insulators under multi-stressed conditions: An overview. Mater. Res. Express.

[2] Abd Rahman R., Harid N., Haddad A. (2010). Stress control on polymeric outdoor insulators. Proceedings of the 45th International Universities Power Engineering Conference UPEC2010.

[3] Schneider H., Guidi W.W., Nicholls C.W., Hall J.F., Burnham J.T. (1992). Accelerated aging chamber for nonceramic insulators. Power Technol. Int..

[4] Schneider H., Guidi W.W., Burnham J.T., Gorur R.S., Hall J.F. (1993). Accelerated aging and flashover tests on 138 kV nonceramic line post insulators. IEEE Trans. Power Deliv..

[5] Ahmad F., Akbar M., Ullah R. (2021). AC performance of HTV-SR and its hybrids loaded with nano-/micro-silica/ATH fillers. Arab. J. Sci. Eng..

[6] Akbar M., Ullah R., Abdul Karim M.R. (2020). Interpreting surface degradation of HTV silicone rubber filled with micro/nano-silica Under AC and DC Voltages. J. Electron. Mater..

[7] Sundararajan R., Olave C., Romero E., Kannan A.M. (2007). Impedance analysis of long term aged thermoplastic elastomeric insulators. Proceedings of the 2007 Annual Report-Conference on Electrical Insulation and Dielectric Phenomena.

[8] Abd-Rahman R., Haddad A., Kamarudin M.S., Yousof M.F.M., Jamail N.A.M. (2016). Dynamic modelling of polluted outdoor insulator under wet weather conditions. Proceedings of the 2016 IEEE International Conference on Power and Energy (PECon).

[9] Ahmed R., Abd Rahman R., Jamal A., Salem A.A., Saman B., Lau K.Y., Ghoneim S.S. (2022). Field-Dependent Pollution Model under Polluted Environments for Outdoor Polymeric Insulators. Polymers.

[10] Ahmed R., Kim T., Lee Y.J., Jeon S., Yi J., Choi I.H., Son J.A., Koo J.B. (2020). Online condition monitoring and leakage current effect based on local area environment. Trans. Electr. Electron. Mater..

[11] Ullah I., Akbar M. (2022). Anti-aging characteristics of RTV-SiR aided HV insulator coatings: Impact of DC polarity and fillers. Mater. Chem. Phys..

[12] Zaghloul M.Y., Zaghloul M.M., Zaghloul M.M. (2022). Influence of stress level and fibre volume fraction on fatigue performance of glass fibre-reinforced polyester composites. Polymers.

[13] Ullah I., Amin M., Nazir M.T., Hussain H. (2020). Impact of accelerated ultraviolet weathering on polymeric composite insulators under high voltage DC stress. CSEE J. Power Energy Syst..

[14] Ullah R., Akbar M. (2021). Effect of AC stressed aging on partial discharge, thermal and tensile performance of silicone rubber-based composites. Compos. Commun..

[15] Ullah R., Akbar M. (2020). Lifetime estimation based on surface degradation and characterization of HTV silicone-rubber based composites for HVAC and HVDC transmission. CSEE J. Power Energy Syst..

[16] Zhu Y., Zhang X., Zhou S., Fang J. (2018). Ageing Performance of HTV Silicone Rubber Used for Outdoor Insulation. Proceedings of the 2018 IEEE/PES Transmission and Distribution Conference and Exposition (T&D).

[17] Salem A.A., Abd-Rahman R., Ahmad H., Kamarudin M.S., Jamal N.A.M., Othman N.A., Ishak M.T. (2018). A new flashover prediction on outdoor polluted insulator using leakage current harmonic components. Proceedings of the 2018 IEEE 7th International Conference on Power and Energy (PECon).

[18] Sundararajan R. (2010). Long term acid rain multistress performance of Thermoplastic and thermoset polymeric insulators. Proceedings of the 2010 5th International Conference on Industrial and Information Systems.

[19] Venkatesulu B., Thomas M.J. (2011). Long-term accelerated weathering of outdoor silicone rubber insulators. IEEE Trans. Dielectr. Electr. Insul..

[20] Ashwini A., Ravi K., Vasudev N. (2019). Experimental Study on Aging of Polymeric Insulators by Dip Method. Proceedings of the 2019 International Conference on High Voltage Engineering and Technology (ICHVET).

[21] Bencherif Y., Mekhaldi A., Lobry J., Olivier M., Poorteman M., Bonnaud L. (2020). Multiscale Analysis of the Polymeric Insulators Degradation in Simulated Arid Environment Conditions: Cross-Correlation Assessment. J. Electr. Eng. Technol..

[22] Verma A.R., Reddy B.S. (2019). Interpretation of surface degradation on polymeric insulators. Eng. Fail. Anal..

[23] Zhu Y., Zhang X., Fang J. (2017). Influence of environmental factor on hydrophobicity transfer of silicone rubber used for outdoor insulation. Proceedings of the 2017 International Symposium on Electrical Insulating Materials (ISEIM).

[24] Liang X., Zhang Y., Yin Y., Li Z. (2012). 5000 h Multi-stress Test Procedure for Silicone Rubber Composite Insulators and Its Applications in Long- term Performance Evaluation. Gaodianya Jishu High Volt. Eng..

[25] Amin S. (2012). Comparative natural aging of thermoplastic elastomeric and silicon rubber insulators in Pakistan. J. Elastomers Plast..

[26] Khattak A., Amin M. (2016). Accelerated aging investigation of high voltage EPDM/silica composite insulators. J. Polym. Eng..

[27] Khattak A., Iqbal M., Amin M. (2017). Aging analysis of high voltage silicone rubber/silica nanocomposites under accelerated weathering conditions. Sci. Eng. Compos. Mater..

[28] Ullah I., Akbar M., Khan H.A. (2021). Degradation analysis of RTV-SiR based composites under both polarities DC voltage for insulators coating. Mater. Today Commun..

[29] Ullah I., Akbar M., Khan H.A. (2022). Enhancement of electrical, mechanical and thermal properties of silicone based coating with aluminatrihydrate/silica for ceramic insulators. Mater. Chem. Phys..

[30] Khan H., Mahmood A., Ullah I., Amin M., Tariq Nazir M. (2021). Hydrophobic, dielectric and water immersion performance of 9000 h multi-stresses aged silicone rubber composites for high voltage outdoor insulation. Eng. Fail. Anal..

[31] Khattak A., Amin M., Ali M., Iqbal M.J. (2020). Hydrophobicity investigation and life estimation of silicone rubber nanocomposites. Eng. Mater. Res..

[32] (2008). Guide for the Selection of Insulators in Respect of Polluted Conditions.

[33] (1992). Hydrophobicity Classification Guide.

[34] Ullah R., Akbar M., Ullah N., Otaibi S.A., Althobaiti A. (2021). Understanding Variations in the Tracking and Erosion Performance of HTV-SR-Based Composites due to AC-Stressed Aging. Polymers.

[35] Sundararajan R., Soundarajan E., Mohammed A., Graves J. (2006). Multistress accelerated aging of polymer housed surge arresters under simulated coastal Florida conditions. IEEE Trans. Dielectr. Electr. Insul..

[36] Sundararajan R., Mohammed A., Chaipanit N., Karcher T., Liu Z. (2004). In-service aging and degradation of 345 kV EPDM transmission line insulators in a coastal environment. IEEE Trans. Dielectr. Electr. Insul..

[37] (2012). Polymeric HV Insulators for Indoor and Outdoor Use—General Definitions, test Methods and Acceptance Criteria.

[38] Yoshimura N., Kumagai S., Nishimura S. (1999). Electrical and environmental aging of silicone rubber used in outdoor insulation. IEEE Trans. Dielectr. Electr. Insul..

[39] Su H., Jia Z., Guan Z., Li L. (2011). Durability of RTV-coated insulators used in subtropical areas. IEEE Trans. Dielectr. Electr. Insul..

[40] Sundararajan R., Olave C., Romero E., Trepanier B. (2007). Modified IEC 5000-h multistress aging of 28-kV thermoplastic elastomeric insulators. IEEE Trans. Power Deliv..

